# Admission deep venous thrombosis of lower extremity after intertrochanteric fracture in the elderly: a retrospective cohort study

**DOI:** 10.1186/s13018-020-02092-9

**Published:** 2020-11-19

**Authors:** Jinzeng Zuo, Yongcheng Hu

**Affiliations:** 1 Graduate School of Tianjin Medical University, No. 406 Jiefang South Road, Tianjin, 300200 P. R. China; 2 Department of Orthopaedic Surgery, The Second Hospital of Tangshan, Tangshan, 063000 Hebei P. R. China; 3 Department of Orthopaedic Oncology, Tianjin Hospital, Tianjin, 300211 P. R. China

**Keywords:** Hip fracture, Deep vein thrombosis, Epidemiology, Risk factors, Individualized risk assessment

## Abstract

**Objective:**

The purpose of this study was to investigate the incidence, location, and related factors of deep venous thrombosis (DVT) of the bilateral lower extremities after intertrochanteric fractures in the elderly.

**Methods:**

Retrospective analysis was performed on the elderly patients with intertrochanteric fracture who were admitted from January 2017 to December 2019. At admission, patients receive routine ultrasound Doppler scanning of bilateral lower extremities to detect DVT; those with DVT were assigned to the case group and those without DVT to the control group. Patient data on demographics, comorbidities, injury-related data, and laboratory test results at admission were extracted. Logistic regression analyses were conducted to identify the independent risk factors associated with DVT.

**Results:**

Five hundred seventy-eight patients were included, among whom 116 (20.1%) had DVT. Among those with DV, 70.7% (82/116) had DVT of the distal type, 24 (29.6%) had DVT of the proximal type, and 10 (10.4%) had mixed DVT. In 76.7% (89/116) of patients, DVT occurred in the fractured extremity, 9.5% (11/116) in the bilateral and 13.8% (16/116) in the non-fractured extremity. Multivariate analyses identified obesity, delay to admission, increased D-dimer level (> 1.44 mg/L) and reduced albumin (< 31.7 g/L) as independent factors.

**Conclusions:**

Admission incidence of DVT was high in elderly patients with intertrochanteric fractures, especially the proximal DVT. Identification of associated risk factors is useful for individualized assessment risk of DVT and early targeted interventions.

## Introduction

Intertrochanteric fracture is one of the most common fractures in the elderly, constituting 10~20% of all fractures and 50–65% of hip fractures [1, 2]. Intertrochanteric fractures are typical osteoporotic fractures and are most commonly caused by low-energy fall injury, with a 1-month mortality of 5–10% and 1-year mortality rate of 8–31% [3]. After an intertrochanteric fracture occurs, the hypercoagulability of blood, post-trauma stress/inflammatory immune response of the body place the patients at a high risk of DVT. It was reported that intertrochanteric fracture itself was a risk factor for DVT, increasing 2.5-fold risk compared to femoral neck fracture [4]. In addition, the high prevalence of multiple comorbidities made elderly patients more susceptible to DVTs than younger patients [5]. Pulmonary embolism (PE) is the third leading cause of death within 24 hours after trauma, and DVT is a most predominant source of PE [6]. Therefore, prompt detect of DVT and targeted anti-thrombotic therapy are of great significance in preventing progress of DVT proximal to PE.

Plasma D-dimer level is the product of plasminozyme mediated degradation of cross-linked fibrin, having a high sensitivity (more than 95%) in diagnosing DVT, but is limited in use due to a poor specificity (only 20–40%) [7]. Despite multiple studies have been performed to identify the risk factors associated with DVT after hip fracture, but most of them were limited by the relatively small sample size or focused on preoperative and postoperative DVTs [8–10], overlooking the clinical importance of screening of DVT at admission. In order to screen DVT more specifically, it is necessary to identify the risk factors associated with admission DVT and their association intensity.

To address this issue, we conducted this study, with aims to investigate the incidence, location, and associated risk factors of admission DVT of bilateral lower extremities in elderly patients with intertrochanteric fracture.

## Materials and methods

This was a retrospective study, approved by the ethics committee of the Tangshan 2nd Hospital. Between January 2017 and December 2019, elderly patients who were admitted at our hospital presenting with intertrochanteric fracture were potentially included in this study.

### Inclusion and exclusion criteria

The inclusion criteria were: patient age of 60 years or older, unilateral intertrochanteric fracture caused by low-energy trauma (fall from standing height), closed fracture, and admitted to hospital within 7 days after injury. The exclusion criteria were high-energy injury, open fracture, subtrochanteric fractures, multiple fractures, history of thromboembolism events, delay to admission (fracture to admission more than 7 days), a past history of hip surgery for any reason, receiving anticoagulant therapy (e.g., aspirin, heparin, low molecular heparin, or other drugs) within 3 months before fracture, incomplete medical records.

### Diagnostic criteria of DVT

DVT was diagnosed according to the Chinese medical association guidelines for the diagnosis and treatment of DVT (3rd edition). Doppler ultrasound scanning is routinely performed at common femoral vein, superficial femoral vein, deep femoral vein, popliteal vein, anterior tibial vein, posterior tibial vein and peroneal vein of bilateral extremities. The diagnostic criteria are (1) the vein cavity cannot not be compressed; (2) solid echo in the cavity; (3) blood flow signal filling defect in the lumen; (4) the enhancement or weakening of the blood flow in the distal extremities. Superficial or isolated calf muscular venous thrombosis was excluded because of their less clinical significance. Popliteal vein and proximal thrombus (superficial femoral vein, deep femoral vein, and common femoral vein) were classified as proximal DVT; anterior or posterior tibial vein, and peroneal vein are classified as distal thrombus. Patients with both proximal and distal thrombus were classified as mixed DVT group.

### Data collection

The inpatient medical record system were used to retrieve the patient’s data, including demographics (age, sex, body mass index (BMI), comorbidities and lifestyles (smoking, drinking, hypertension, diabetes, congestive heart disease, lung disease, liver disease, kidney disease, rheumatoid and connective tissue disease, peripheral vascular disease), injury-related characteristics (time from injury to admission, fracture type based on AO/OTA classification system, and laboratory test results at admission (platelet, fasting blood glucose (FBG), total protein (TP), albumin (ALB), total cholesterol (TC), triglyceride (TG), red blood cell count (RBC), hemoglobin (HGB), white blood cell (WBC), neutrophil (NEUT), lymphocyte (LYM), plasma D-dimer levels).

### Statistical analysis

Patients were divided into DVT and non-DVT group. Continuous variables were expressed by mean and standard deviation (SD) and were evaluated by Student’s *t* test or Whitney *U* test. The categorical variables were expressed as number (percentage) and were evaluated by chi-square or Fisher’s exact test.

Considering the significance of D-dimer level and ALB level in clinical practice, receiver operating characteristic (ROC) curve was used to determine the optimal cut-off values, namely when the Youden index (sensitivity + specificity − 1) was maximum. The traditionally used cut-off value for them was also evaluated.

Variables tested to be approximately significant (*p* < 0.1) in the univariate analyses were incorporated in the multivariate model, using step-back elimination method, to determine the independent risk factors. Variables with *p* less than 0.10 were retained in the final model, and the odd ratio (OR) indicated the correlation strength. *p* < 0.05 was set as statistical significance level. SPSS 22.0 was used to perform all the analyses (SPSS, IBM, Armonk, New York, USA).

## Results

A total of 578 patients with intertrochanteric fractures were included, including 217 males and 361 females, with an average age of 76.6 ± 8.7 years (range, 60~102 years). According to the AO/OTA classification system, there were 146 A1 type, 340 A2 and 127 and 92 A3 type. The average length of the patients from injury to admission was (1.24 ± 1.78 days).

A total of 116 patients were diagnosed to have DVT, with an incidence of 20.1%. Among 116 patients with DVT, 70.7% (82/116) had distal DVT, 24 (20.7%) had proximal and 10 (8.6%) had mixed DVT, with the incidence being 14.2%, 4.2%, and 1.7%, respectively. In 76.7% (89/116) of patients, DVT occurred in the fractured extremity, 9.5% (11/116) in the bilateral and 13.8% (16/116) in the non-fractured extremity. A total of 187 thrombi were found in 116 patients, with an average of 1.6 thrombus per patient. Patients diagnosed with DVT had no complained clinical DVT symptom, and none of them developed pulmonary embolism.

**Fig. 1:**
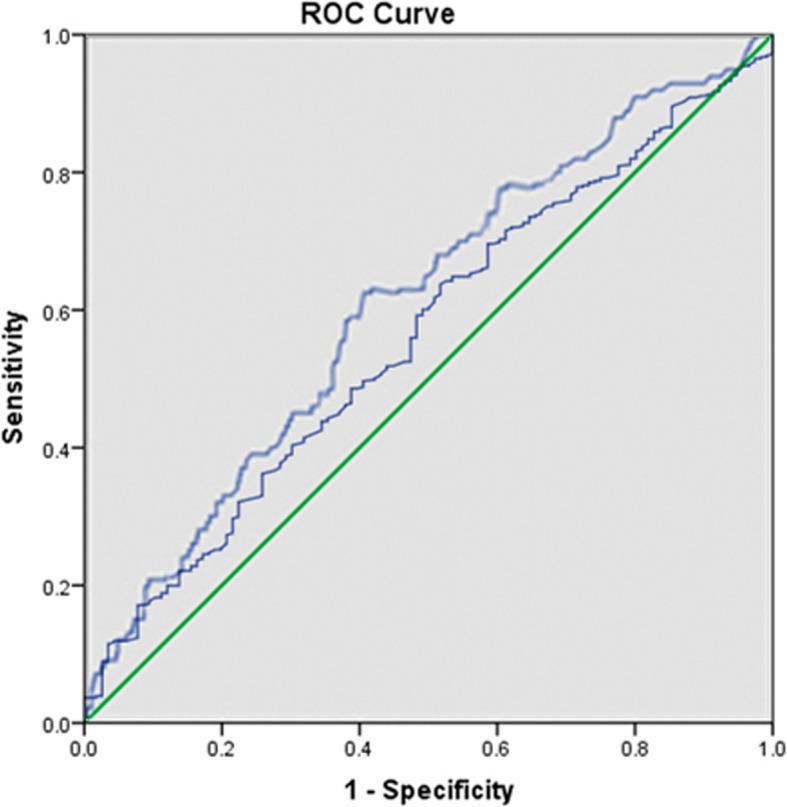
The optimal cut-off value of D-dimer level and ALB was determined by ROC curve to be 1.44 mg/L (sensitivity, 0.680; specificity, 0.478) and 31.7 g/L (sensitivity, 0.638; specificity, 0.543), respectively

ROC curve showed that the optimal cut-off value of D-dimer was 1.44 mg/L (Fig. 1), at which the sensitivity was 68.0% and the specificity was 47.8%, and the area under the curve was 0.60 (95% CI, 0.54 to 0.66) with statistical significance (*p* = 0.002). The optimal cut-off value of ALB was 31.7 g/L, at which sensitivity was 63.8% and the specificity was 54.3%, and the area under the curve was 0.56 (95% CI 0.51 to 0.62) with statistical significance (*p* = 0.039).

There was significant difference between two groups in term of BMI both in continuous and categorical variable, time from injury to admission, ALB (31.7 g/L as cut-off value), NEUT, RBC, HGB, and D-dimer (> 1.44 mg/L) (all *p* < 0.05) (Table 1). The multivariate regression analysis showed that obesity, delay to admission, increased D-dimer level (> 1.44 mg/L), and reduced albumin (< 31.7 g/L) were identified as independent factors associated with DVT (Table 2).

**Table 1:** Comparison between DVT and non-DVT patients with intertrochanteric fractures

Variable	DVT group (***n*** = 116)	Non-DVT group (***n*** = 462)	***p*** value
Gender (male)	41 (35.3)	173 (37.4)	0.675
Age (years)	77.5 ± 8.9	76.5 ± 9.4	0.687
60–69	27 (23.3)	113 (24.5)	0.965
70–79	40 (34.5)	157 (34.0)	
80 and above	49 (42.2)	192 (41.6)	
BMI (kg/m) ^2^	24.5 ± 4.2	23.4 ± 3.3	0.003
< 18.5	7 (6.0)	27 (5.8)	0.005
18.5–23.9	51 (44.0)	252 (54.5)	
24.0–27.9	36 (33.6)	139 (30.1)	
28.0 or higher	22 (18.4)	41 (8.9)	
Smoking	16 (13.8)	62 (13.4)	0.899
Diabetes	29 (25.0)	125 (27.1)	0.654
Hypertension	72 (62.1)	271 (58.7)	0.504
Cerebrovascular disease	40 (34.5)	166 (35.9)	0.771
Chronic heart disease	39 (33.6)	150 (32.5)	0.813
History of lung disease	6 (5.2)	27 (5.8)	0.780
Liver disease	10 (8.6)	37 (8.0)	0.829
Kidney disease	6 (5.2)	22 (4.8)	0.854
Rheumatoid and connective tissue disease	5 (4.3)	20 (4.3)	0.993
Peripheral vascular disease	9 (7.8)	26 (5.6)	0.390
Allergy	24 (20.7)	101 (21.9)	0.878
ASA			0.584
I	7 (6.0)	25 (5.4)	
II	51 (44.0)	228 (49.4)	
III	58 (50.0)	209 (45.2)	
Fracture classification (AO/OTA)			0.413
A1	29 (25.0)	117 (25.3)	
A2	73 (62.9)	267 (57.8)	
A3	14 (12.1)	78 (16.9	
Interval between injury and admission (d)	1.8±2.6	1.1±2.3	0.002
TP (< 58 g/L)	63 (54.3)	207 (44.8)	0.067
ALB (< 31.7 g/L)	55 (47.4)	165 (35.7)	0.020
ALB (< 35 g/L)	83 (71.6)	290 (62.8)	0.077
LDH (> 250 u/L)	34 (29.3)	133 (28.8)	0.912
TC (> 5.2 mmol/L)	17 (14.7)	42 (9.1)	0.077
TG (> 1.7 mmol/L)	13 (11.2)	34 (7.4)	0.175
HDL-C (< 1.1 mmol/L)	59 (50.9)	174 (37.7)	0.010
FBG (> 6.1 mmol/L)	72 (62.1)	243 (52.6)	0.067
NEU (> 6.3 × 10^9^/L)	66 (56.9)	212 (45.9)	0.034
LYM (< 1.1 × 10/L) ^9^	59 (50.9)	237 (51.3)	0.933
RBC (< lower limit)	77 (66.4)	245 (53.0)	0.010
HGB (< lower limit)	81 (69.8)	259 (56.1)	0.007
PLT (> 286 × 10^9^/L)	39 (33.6)	132 (28.6)	0.287
PDW			0.631
12~18.1%	97 (90.4)	392 (89.6)	
< 12%	15 (8.0)	48 (7.5)	
> 18.1%	4 (1.6)	22 (2.9)	
D-dimer (> 0.5 mg/L)	93 (80.2)	377 (81.6)	0.724
D-dimer (> 1.44 mg/L)	68 (58.6)	214 (41.3)	0.018

*Note: DVT* deep vein thrombosis, *BMI* body mass index, *TP* total protein, *ALB* serum albumin, *LDH* lactate dehydrogenase, *TC* total cholesterol, *TG* triglyceride, *HDL* high-density lipoprotein, *Na+* sodium concentration, *FBG* fasting blood glucose, *RBC* red blood cells, reference range: male, 4.0~5.5 × 10^12^/L, female, 3.5~5.0 × 10^12^/L. *HGB* hemoglobin, male, ≥ 120 g/L, female, ≥ 110 g/L, *WBC* white blood cell, *NEUT* neutrophils, *LYM* lymphocyte, *PLT* platelets, *PDW* platelet distribution width

**Table 2:** Multivariate analysis of admission DVT in elderly patients with intertrochanteric fracture

Variable	OR and 95% CI	***p***
BMI (kg/m)^2^		
18.5–23.9	Reference	
< 18.5	1.07 (0.78–1.19)	0.734
24.0–27.9	1.28 (0.81–2.05)	0.299
≥ 28.0	2.93 (1.60–5.36)	< 0.001
Time from injury to admission (day)	1.37 (1.06–1.87)	0.003
ALB < 31.7 g/L	1.73 (1.14–2.64)	< 0.001
D-dimer > 1.44 mg/L	1.76 (1.16–2.68)	0.009

*Note*: *DVT* deep vein thrombosis, *BMI* body mass index, *ALB* albumin

## Discussion

In elderly patients with trauma, it remains an intractable issue that 15 to 48% of patients had perioperative DVT, even they were given thromboprophylaxis [4, 11]. The present study focused on admission DVT following intertrochanteric fractures, and found the incidence of 20.1%, and particularly that of proximal thrombosis being 5.9%; obesity, delay to admission, increased D-dimer level (> 1.44 mg/L) and reduced albumin (< 31.7 g//L) were identified as independent factors associated with DVT.

Currently, most studies focused on the preoperative or postoperative DVT, and less attention is paid to the DVT that may have occurred at the time of admission. In this study, 20.1% of elderly patients had DVT upon their admission, which was comparable to that of literature. Lu et al. [12] found the incidence rate of DVT was 15.8% following hip fracture, lower than ours, which may be explained by the much younger age (median, 39.6 years, interquartile rang of 28–50 year) of patients in their study. Xia et al. [13] reported the preoperative DVT developed in 18.9% of the 301 elderly patients with femoral neck fracture. However, in their study, isolated calf muscular venous thrombosis had also been considered as DVT, and indeed it took an extraordinary proportion (77.2%). In another study, Xing et al. [5] reported a higher prevalence of DVT following hip fracture in the elderly at admission, that was 29.8%, but they did not specify whether calf muscular venous thrombosis had been excluded.

It should be noted that the all the DVTs confirmed in this study were asymptomatic, that is, occult thrombus, therefore increasing the difficulty of screening. However, the increasing evidences have showed no difference in cause of pulmonary embolism and even death between occult and symptomatic DVTs [11], and therefore they should be equally medically treated. Accordingly, routine screening of DVT is necessarily performed at admission for elderly patients with intertrochanteric fractures, and those confirmed to have DVT should be immediately managed with thrombolytic therapy rather than prophylaxis in general. In our study, the incidence of proximal and mixed thrombus was 5.9%, in range of reported figures of 3.6–8.8% [5, 10, 13]. The studies have demonstrated that 4–11% of the proximal thrombus would progress to lead to pulmonary embolism [14], which further highlighted the importance of screening of DVT upon at admission.

Most, but not all the studies demonstrated the advanced age to be an independent risk factor for DVT after major trauma. Yeol et al. [15] reported the incidence of DVT after major lower limb orthopedic surgery and found 5-fold increased risk in patients of 50–69 years and 10-fold higher in those of 70 years above, compared to those aged < 49 years. Dong et al. [9] demonstrated in a retrospective study of 534 traumatic fractured patients that age above 60 years was an independently associated factor for perioperative DVT. However, in some studies specifying elderly patients with trauma, including but not limited to hip fracture, most studies failed to demonstrate a significant relationship between age and DVT [8, 10, 13], similar as the present study. It is likely that there exists a cut-off value of age above which the risk of DVT increases significantly, and this value should be at a relatively younger age rather than an elderly age. On the other hand, the higher prevalence of comorbidities or relatively poorer vascular pathophysiology among elderly patients might also contribute to the DVT. These speculations have plausibility to some extent, but require to be verified in the further study.

The positive relationship between delay to admission and DVT has been consistently a research focus, and this study demonstrated every delay of 1 day to admission was associated with 37% increased risk of DVT. In a previous study, Li et al. [4] studied 1183 elderly hip fractures and found early admission (within 24 h after injury) was associated significantly lower incidence of perioperative DVT than delay to admission (21.9% verse 35.7%). Other researchers also got the similar findings, either for elderly hip fractures or other traumatic fractures [5, 9]. This could be explained by the hypercoagulability of blood with time and body’s post-trauma stress, both of which are at dynamic peak within 1–4 days after trauma [16]. Therefore, early screening for DVT and operation performed within 24–48 h after injury should provide a feasibility to reduce the perioperative adverse events [17]. Additionally, for patients who have delayed admission above 3 days, more attention should be paid to avoid missed diagnosis of DVT.

Plasma D-dimer level is an important indicator of fibrinolytic activity, which reflects the hypercoagulability and fibrinolytic activity, with high sensitivity of 90~100% but low specificity in diagnosis of DVT. But it is susceptible to a variety of factors, including age [18] and post-trauma stress [19]. In this study, the optimal cut-off value of D-dimer level was determined to be 1.44 mg/L, above which the risk of DVT significantly increased, independent of multiple variables. However, its specificity remains low, being 47.8%, which could be used as an auxiliary factor to improve diagnostic accurate rate. The reduced serum albumin is common in elderly patients with hip fracture [20, 21], and in this study 64.5% of patients had albumin less than 35 g/L. For clinical utility purpose, we determined the cut-off value to be 31.7 g/L, which was more specified in diagnosing DVT (specificity, 54.3%) and therefore was practical in clinical practice.

As with previous studies [9, 10, 22], this study confirmed the adverse effect of obesity on DVT, and the mechanism may be hyperlipidemia and secondary atherosclerosis in obese patients. Kornblith et al. [23] demonstrated obesity independently contributed to hypercoagulability after injury. Additionally, the functional exercise and activity in obese patients were less than that of non-obese patients, which increases the pressure of venous valve and the risk of hemodynamic abnormalities [24]. Furthermore, obesity demonstrated to be associated with more surgical duration, intraoperative blood loss, postoperative infection, and even mortality [25]. Therefore, obese patients should be particularly paid more attention to, such as timely and multiple screenings for avoidance of missed diagnosis, prescribed anti-thromboembolic agents based on body weight, and individualized physiothreaphy program.

Several limitations in this study should be noted. First, the retrospective design has its inherent limitation of accuracy in data collection. Second, as every other multivariate analysis, we could not include all confounding factors and the residual confounding remains an issue. Third, for some key variables that have important effect on DVT development were not available, including the duration of the injured limb. Fourth, this was a single trauma-center study, and the results might not be generalizable. Fifth, the multivariate analyses yeilded the association retionship between variables and DVT, rather than causative relationship.

In summary, the incidence of admission DVT following intertrochanteric fractures in the elderly was 20.1%. Several risk factors were independently associated with DVT, including obesity, delay to admission, D-dimer > 1.44 mg/L, and reduced albumin level. These epidemiologic data are helpful in assessment and stratification risk of DVT, and guiding the subsequent individualized intervention program.

## Data Availability

All the data will be available upon motivated request to the corresponding author of the present paper

## Abbreviations

DVT: Deep venous thrombosis
PE: Pulmonary embolism
DUS: Duplex ultrasonography
FBG: Fasting blood glucose
BMI: Body mass index
TP: Total protein
ALB: Albumin
TC: Total cholesterol
TG: Triglyceride
AUC: Area under the curve
RBC: Red blood cell
WBC: White blood cell
HGB: Hemoglobin
NEUT: Neutrophil
LYM: Lymphocyte
OR: Odd ratio
95% CI: 95% confidential interval
SD: Standard deviation
ROC: Receiver operating characteristic
